# Beneficial Shifts in the Gut Bacterial Community of Gilthead Seabream (*Sparus aurata*) Juveniles Supplemented with *Allium*-Derived Compound Propyl Propane Thiosulfonate (PTSO)

**DOI:** 10.3390/ani12141821

**Published:** 2022-07-17

**Authors:** Miguel Rabelo-Ruiz, Antonio M. Newman-Portela, Juan Manuel Peralta-Sánchez, Antonio Manuel Martín-Platero, María del Mar Agraso, Laura Bermúdez, María Arántzazu Aguinaga, Alberto Baños, Mercedes Maqueda, Eva Valdivia, Manuel Martínez-Bueno

**Affiliations:** 1 Departamento de Microbiología, Universidad de Granada, Avda. Fuentenueva, s/n, 18071 Granada, Spain; mrabelo@correo.ugr.es (M.R.-R.); antnewpor@ugr.es (A.M.N.-P.); ammartin@ugr.es (A.M.M.-P.); mmaqueda@ugr.es (M.M.); evavm@ugr.es (E.V.); 2 Aquaculture Technology Centre of Andalusia, CTAQUA. Muelle Comercial s/n, El Puerto de Santa María, 11500 Cádiz, Spain; mm.agraso@ctaqua.es (M.d.M.A.); lau.ber@hotmail.es (L.B.); 3 Departamento de Microbiología y Biotecnología, DMC Research Center, Camino de Jayena s/n, 18620 Granada, Spain; arancha.aguinaga@domca.com (M.A.A.); abarjona@dmcrc.com (A.B.); 4 Instituto de Biotecnología, Universidad de Granada, 18071 Granada, Spain

**Keywords:** *Allium*-based phytogenic, body weight, gilthead seabream, *Sparus aurata*, gut microbiota, propyl propane thiosulfonate

## Abstract

**Simple Summary:**

Aquaculture plays an important role in supplying global food demand and protein sources. The increasing restriction of drugs in fish production has forced this sector to carry out changes in the management of farms. Functional feed additives such as probiotics, prebiotics, and phytogenics have been proposed in order to maintain or improve productive levels and general health status of fish. In this study, we explore the effects of *Allium*-derived food additives in the bacterial community and growth of gilthead seabream (*Sparus aurata*) juveniles. We found that this additive produced significant changes in bacterial community of the hindgut. In this sense, this shift occurred towards a more diverse microbiota. Especially relevant is the decrease in the populations of potential pathogenic bacteria as *Vibrio* and *Pseudomonas*, while this additive enhanced *Lactobacillus*, a well-known beneficial genus. Our work shows that the addition of PTSO has beneficial effects on bacterial communities while keeping productive parameters on fish growth.

**Abstract:**

This study analyzes the potential use of an *Allium*-derived compound, propyl propane thiosulfonate (PTSO), as a functional feed additive in aquaculture. Gilthead seabream (*Sparus aurata*) juveniles had their diet supplemented with this *Allium*-derived compound (150 mg/kg of PTSO) and were compared with control fish. The effects of this organosulfur compound were tested by measuring the body weight and analyzing the gut microbiota after 12 weeks. The relative abundance of potentially pathogenic *Vibrio* and *Pseudomonas* in the foregut and hindgut of supplemented fish significantly decreased, while potentially beneficial *Lactobacillus* increased compared to in the control fish. Shannon’s alpha diversity index significantly increased in both gut regions of fish fed with a PTSO-supplemented diet. Regarding beta diversity, significant differences between treatments only appeared in the hindgut when minority ASVs were taken into account. No differences occurred in body weight during the experiment. These results indicate that supplementing the diet with *Allium*-derived PTSO produced beneficial changes in the intestinal microbiota while maintaining the productive parameters of gilthead seabream juveniles.

## 1. Introduction

Aquaculture in general plays an important role in supplying the global food demand and protein sources, with fish accounting for 17% of the world’s animal protein intake. In 2018, world aquacultural production reached 82 million tons, with an estimated value of USD 250 billion [1]. However, fish diseases caused by several pathogenic bacteria negatively affect economic profits in the industry. The majority of severe infectious diseases in the industry include furunculosis, photobacteriosis, and vibriosis, caused by *Aeromonas*, *Photobacterium,* and *Vibrio* species, respectively [2]. The prevention of these diseases involves the use of subtherapeutic doses of antibiotics, which increase the risk of the appearance of resistant bacteria, a serious public health problem. In recent years, the use of antibiotics in this sector has been considerably reduced [3]. Faced with this situation, the aquacultural sector demands new alternatives that improve the health status of animals, prevent the appearance of infectious processes, and hence reduce the use of drugs. These alternatives include good management practices such as reducing animal density, vaccinations, phagotherapy, and nutritional interventions that include the use of functional additives such as probiotics, prebiotics, synbiotics, and phytogenics [4,5,6,7,8].

In this context, Annex 1 of Regulation no. 1831/2003 (EC) establishes various categories of feed additives, including zootechnical agents [9]. Within this category, the regulation establishes several functional groups that include digestibility enhancers and intestinal flora stabilizers, described as microorganisms or other chemically defined substances that, when provided to animals, have a positive effect on the intestinal microbiota. Phytogenics are included in this category, defined as plant-derived bioactive compounds supplemented in the diet to improve the productivity or health status of livestock [10]. These include a wide range of plant-derived products such as essential oils, extracts, and oleoresins. Phytogenics contain active ingredients with interesting functional properties such as antimicrobial activity, avoiding the adhesion of pathogens to intestinal mucosa and modulating gut microbiota [11,12]. Therefore, phytogenics can minimize the risk of development of pathogens [12], and the changes in digestive function induce the growth of beneficial bacteria such as *Lactobacillus* or bifidobacteria [13,14].

*Allium*-species plants, mainly garlic (*Allium sativum*) and onion (*Allium cepa*), produce a wide variety of organosulfur compounds showing antifungal, antimicrobial, antioxidant, antiparasitic, and antiviral activity [15,16], and beneficial effects on the immune system [17,18]. These benefits were also observed in several studies in aquaculture, improving immune function, blood parameters, and health status in various fish species [19,20,21,22,23]. The activity of these plant compounds in animal feed is related to secondary metabolites, volatile organosulfur compounds such as ajoene, allicin, isoalliin, propyl propane thiosulfinate (PTS) or propyl propane thiosulfonate (PTSO) [23,24]. In particular, PTSO, a chemically defined molecule, is widely reported for its antibacterial, antifungal, and anticoccidial activity [25,26,27]. It showed beneficial effects on the gut health and changes in the gut microbiota of different terrestrial animal species, such as broiler chickens, laying hens, and pigs [28,29,30,31,32,33,34]. In addition, PTSO showed a significant anti-inflammatory effect in colitis mouse models associated with a modulation of the intestinal microbiota [35]. PTSO was toxicologically safe in studies carried out in these experimental animals [36,37,38,39].

Considering the antecedents of this compound in the modulation of the gut microbiota in terrestrial animals, and the beneficial implications in the health status of animals, we hypothesize that the addition of PTSO in fish diet has a positive effect in gut microbiota. To the best of our knowledge, this effect has not been evaluated yet. Therefore, in this work, we studied the influence of this *Allium*-derived PTSO in foregut and hindgut microbiota by high-throughput sequencing of the 16S rRNA in gilthead seabream (*Sparus aurata*) juveniles.

## 2. Materials and Methods

### 2.1. Allium-Based Product

The *Allium*-based product used is commercialized under the trademark AquaGarlic^®^ and was supplied by DOMCA (Granada, Spain). This product is standardized in propyl propane thiosulfonate (PTSO) at a concentration of 10%. It is in powder form supported on inert sepiolite.

### 2.2. Animals, Experimental Design, and Sample Collection

This research was carried out with gilthead seabream juveniles. Animals (*n* = 780) were randomly assigned to two different experimental groups (390 fish per group) consisting of three tanks per treatment (400 L per tank; 130 fish per tank). Fish were kept in a recirculating aquaculture system D-400 water system equipped with physical and biological filters. The temperature was maintained at 21 ± 1 °C with a photoperiod regime of 12:12 h (light:dark).

The experimental diet was produced from commercial fish meal (NUTRAPLUS, Dibaq, Spain) by adding the *Allium*-based product (1.5 g/kg; final PTSO concentration: 150 mg/kg). Once the meal had been homogenized, the granulated fish feed was manufactured by SPAROS (Olhão, Portugal). A diet without additive was prepared as a control. In addition, to ensure the concentration of PTSO in the feed, UHPLC–ESI–MS/MS analyses were performed according to Abad et al. [40]. The concentration of PTSO in the fish feed was 138 ± 5.32 mg/kg, with an extraction yield of 92% of the analytical method, thus confirming a correct level of inclusion of the active ingredient.

Before the beginning of the trial, fish were randomly housed in different tanks receiving the same initial biomass in each tank. After 2 weeks of acclimatization, fish were anesthetized with 80 mg/L of tricaine methanesulfonate (MS-222) and weighed, with an average initial body weight (BW) of 7.72 ± 0.61 g. During the experiment (12 weeks), fish were fed ad libitum 3–4 times per day, 6 days per week. All the fish from each tank were collected every 2 weeks until the end of the experiment, anesthetized using MS-222, and weighed in groups of 10 fish. At the end of the experiment (12 weeks), 20 fish per experimental tank were euthanized by an overdose of MS-222 (400 mg/L) followed by spine severing. The fish were immediately dissected, and the whole intestine was collected with sterile material. Intestinal pieces were stored in sterile containers and transported to the laboratory, where they were kept at −80 °C until DNA extraction.

### 2.3. DNA Extraction

Intestinal pieces from the foregut and hindgut of gilthead seabream juveniles were dissected using a sterile scalpel, and approximately 100 mg of gut was crushed using a FastPrep FP120 cell disrupter (BIO 101, Thermo Savant, Irvine, CA, USA). DNA extraction was carried out using FavorPrep™ Stool DNA Isolation Mini Kit (Favorgen Biotech Corp., Taipei, Taiwan) according to the manufacturer’s instructions. DNA extraction was checked with 0.7% agarose gel electrophoresis, and DNA concentration was measured using NanoDrop™ 2000 Spectrophotometer (Thermo Fisher Scientific, Waltham, MA, USA). Samples were stored at −20 °C until DNA amplification.

### 2.4. 16S rRNA Gene High-Throughput Sequencing

Amplicon PCR was performed on the bacterial total DNA of the V4 region of the 16S rRNA gene using forward primers U515F (5’ TCGTCGGCAGCGTCAGATGTGTATAAGAGACAGGTGCCAGCMGCCGCGGTAA-3´) or U515F_bar2 (TCGTCGGCAGCGTCAGATGTGTATAAGAGACAGACGAAGTGCCAGCMGCCGCGGTAA); and the reverse primer E786R (5´- GTCTCGTGGGCTCGGAGATGTGTATAAGAGACAGGGACTACHVGGGTWTCTAAT-3´) with Illumina adapter overhang sequences as indicated by the underlined text. Then, a second PCR was performed in order to add two unique Illumina compatible barcodes to each sample, so that the derived sequences could be demultiplexed into their respective samples in downstream analysis (Table S1). These barcodes overlapped with the sequences of the primers used in the first PCR. Purification steps were performed using DNA Purification SPRI Magnetic Beads (Canvax^®^, Cordoba, Spain). PCR amplicons were checked by 1% agarose gel electrophoresis. DNA concentrations were measured using Qubit^®^ 3.0 Fluorometer (Invitrogen™, Carlsbad, CA, USA) and normalized to reach the same concentration per sample. High-throughput sequencing was performed using Nextera XT DNA Library Prep Kit (Illumina, San Diego, CA, USA). This sequencing resulted in paired-end reads of 2 × 300 bp length. Sequencing was carried out on the Illumina MiSeq platform in the Scientific Instrumental Center at the University of Granada (CIC-UGR, Granada, Spain).

### 2.5. Sequences Processing and Data Analysis

Quantitative Insights Into Microbial Ecology, QIIME2 v2020.11 [41] software was used to analyze the 16S rRNA sequences generated from Illumina MiSeq. First, primer trimmings were performed using the *cutadapt* plugin [42]. Reverse reads resulted in low quality, and paired joining retained a low percentage of the original sequences (<10%). Therefore, we proceeded with forward reads in the following analysis. Quality filtering was performed using a Phred score of 20 as the threshold. Deblur was used for sequence clustering into Amplicon Sequence Variants (ASVs) in order to remove sequencing errors [43]. Sequences that passed the quality filters were trimmed to 200 bp, giving a dataset of 5,568,329 total reads with a mean of 21,667 reads per sample. The fragment insertion script implemented in QIIME2 was used to align the sequences and build a bacterial phylogenetic tree on the basis of reference phylogenetic tree SEPP reference Greengenes 13.8 [44]. Taxonomy assignation was based on a classifier pretrained on Greengenes 13.08 with a similarity of 99% [45]. Lastly, reads of chloroplasts and mitochondria were excluded by filtering the ASV table.

### 2.6. Statistics

To test the effect of treatment on the fish’s body weight, we used generalized linear mixed models (GLMMs). We used the body weight of 10 fish as the dependent variable with treatment as a fixed factor, sampling time as a covariate, and tank nested in treatment as a random factor. No differences appeared in initial body weight between the control and *Allium*-supplemented fish (GLMM, initial BW as dependent variable, treatment as fixed factor: control: 78.93 ± 0.67, treatment: 77.96 ± 0.62, F_1,72_ = 3.12; *p* = 0.268; tank nested in treatment as random factor, F_4,72_ = 1,29; *p* = 0.622).

For alpha and beta diversity analyses, the ASV table was rarified at 8000 sequencing depth per sample. Samples that did not reach this sequencing depth were excluded from subsequent analyses. Sample size was 48 for control foregut, 55 for control hindgut, 42 for *Allium*-supplemented-foregut, and 51 for *Allium*-supplemented-hindgut.

Two alpha diversity indices were calculated, i.e., Shannon diversity index (Shannon, 1948) [46]; and bacterial ASV richness (or number of observed ASVs). We used GLMM to explore the effect of treatment and gut region as fixed factors, and tank nested in treatment as a random factor in both alpha diversity indices. In these analyses, fish were the experimental unit for alpha and beta diversity analysis. Body weight and alpha diversity analyses were performed using STATISTICA 10.0 (StatSoft).

Differences in genus and class abundances between control and treated fish were explored by means of linear discriminant analysis effect size (LEfSe) [47]. LEfSe analyses were performed on the Galaxy web platform, implemented in a public server [48] (https://huttenhower.sph.harvard.edu/galaxy/, accessed on 4 July 2022).

Beta diversity distance matrices were calculated using UniFrac distance. Both weighted and unweighted UniFrac indices [49,50] were used for subsequent analysis. Weighted UniFrac gives more importance to most abundant ASVs, while unweighted UniFrac gives more importance to low-abundance ASVs, as it takes their presence or absence irrespective of their abundance. Permutational ANOVA (PERMANOVA) included these abundance matrices as dependent matrices, treatment and gut region as fixed factors, and tank nested in treatment as a random factor. PERMANOVA was performed in PRIMER-7 software (PRIMER-e) with a PERMANOVA plugin implemented. Principal coordinate analyses (PCoA) were performed in order to visualize the first two axes using EMPeror 2018.2.0 [51].

## 3. Results

### 3.1. Changes in Bacterial Community Composition

**Figure 1:**
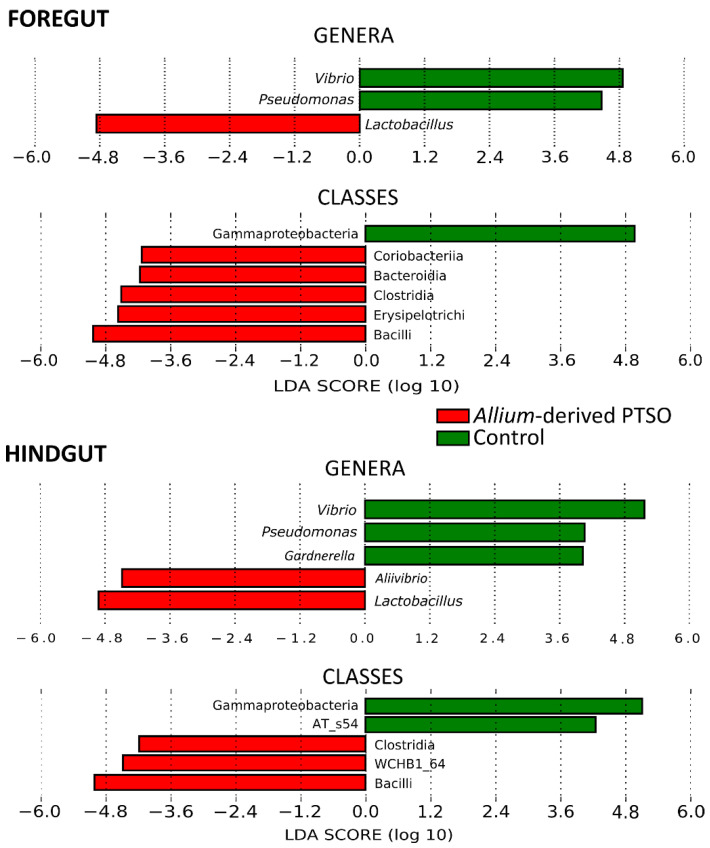
LDA effect size (LEfSe) analyses showing bacterial classes and genera that differed significantly between control fish and those supplemented with *Allium*-derived PTSO in the foregut and hindgut. Significant LDA score > 4.0. Sample size was 48 for control foregut, 55 for control hindgut, 42 for *Allium*-supplemented foregut, and 51 for Allium-supplemented hindgut.

The gut microbiota of the gilthead seabream juveniles was dominated by classes Gammaproteobacteria, Bacilli, and Actinobacteria. The relative abundance of these classes depended on the gut region and treatment. Gammaproteobacteria showed higher abundances in the control group in both gut regions, while Bacilli significantly dominated both gut regions in the *Allium*-supplemented group (Figure 1, Supplementary Figure S1).

Furthermore, significant differences appeared in the minority classes. In the foregut, Coriobacteriia, Bacteroidia, Clostridia, and Erysipelotrichi showed higher abundance in the Allium-supplemented group. In the hindgut, Clostridia and class WCHB1_64 (included in the candidate phylum OP11) were higher in *Allium*-supplemented group, while class AT_s54 was higher in the control group (Figure 1).

**Figure 2:**
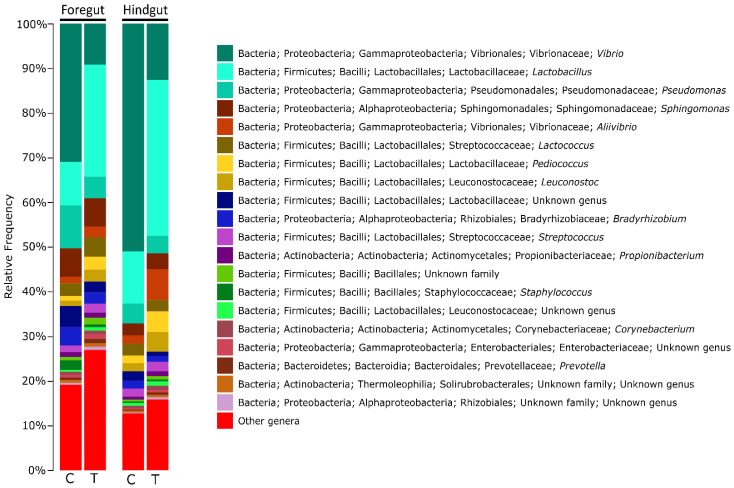
Bar plot summarizing the relative bacterial abundance at the genus level in different gut regions (foregut and hindgut) and treatments. Control (C) refers to gilthead seabream juveniles fed with basal diet while *Allium*-derived PTSO (T) refers to experimental gilthead seabream juveniles fed with basal diet supplemented with *Allium*-derived PTSO. Sample size was 48 for control foregut, 55 for control hindgut, 42 for *Allium*-supplemented foregut, and 51 for *Allium*-supplemented hindgut.

At the genus level, the foregut and hindgut of control fish were dominated by *Vibrio*, *Pseudomonas*, *Lactobacillus*, and *Sphingomonas*. These genera were also the most abundant in the *Allium*-supplemented group, but the relative abundance of these genera changed compared to in the control group (Figure 2). While *Vibrio* and *Pseudomonas* showed significantly higher abundances in the foregut of control group, *Lactobacillus* abundance was significantly higher in the foregut of *Allium*-supplemented group (Figure 1). In the hindgut, genera *Vibrio* and *Pseudomonas*, and minority genus *Gardnerella* also showed significantly higher abundances in control group, while the Allium-supplemented group experienced significantly higher abundances of *Lactobacillus* and *Allivibrio* (Figure 1).

### 3.2. Effect of Allium-Derived PTSO Supplementation on Alpha and Beta Diversity Indices

**Figure 3:**
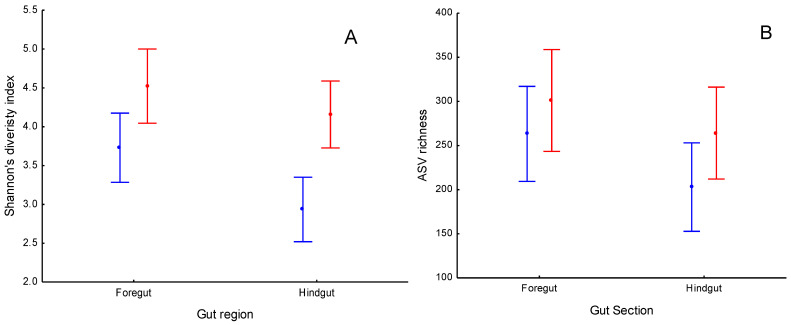
(**A**) Shannon’s diversity index and (**B**) ASV richness (number of bacterial ASV) of bacterial community of foregut and hindgut of gilthead seabream juveniles fed with control (blue) and *Allium* supplemented diets (red). Sample size was 48 for control foregut, 55 for control hindgut, 42 for *Allium*-supplemented foregut, and 51 for *Allium*-supplemented hindgut.

Supplementing the diet of gilthead seabream juveniles with *Allium*-derived PTSO affected the Shannon diversity index in both gut regions (Table 1, Figure 3A). The *Allium*-supplemented group showed higher diversity than the control did in the foregut and hindgut (LSD post hoc test, *p* < 0.012, Figure 3A). However, no differences appeared in the number of bacterial ASV richness between the control and *Allium*-supplemented groups in either the foregut or the hindgut (Table 1; LSD post hoc test; *p* > 0.107, Figure 3A). Shifts in bacterial alpha diversity between the foregut and hindgut were similar between the control and *Allium*-supplemented group (see Gut Region*Treatment interaction term in both alpha diversity indexes in Table 1, Figure 3A,B).

**Figure 4:**
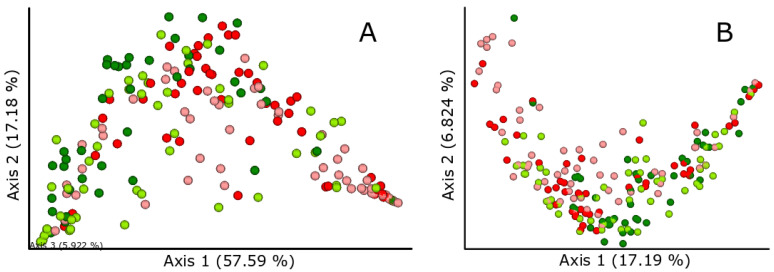
Principal coordinate analysis based on (**A**) weighted and (**B**) unweighted UniFrac distance matrices exploring the effects in the bacterial gut community of the supplementation with *Allium*-derived PTSO in the diet of gilthead seabream juveniles (red: foregut—control fish; pink: hindgut—control; green: foregut—treated fish; light green: hindgut—treated fish). Percentages show the proportion of variance explained by each axis. Sample size was 48 for control foregut, 55 for control hindgut, 42 for *Allium*-supplemented foregut, and 51 for *Allium*-supplemented hindgut.

The bacterial community of gilthead seabream juveniles did not significantly vary between the two diets, when taking into account either the most abundant bacterial ASVs (weighted UniFrac) or minority ASVs (unweighted UniFrac) (Table 2, Figure 4). In the foregut, marginally significant differences between treatments appeared using weighted UniFrac. Similar nonsignificant trends were found in the hindgut for both unweighted and weighted UniFrac (Table 2). The bacterial community in the foregut significantly differed from the hindgut microbiota irrespective of treatment (Table 2, Figure S2). In fact, shifts in the bacterial community between the foregut and hindgut showed similar trends in the control and *Allium*-supplemented group (see nonsignificant Gut Region*Treatment interaction terms in Table 2).

Significant differences appeared between tanks in the same treatment group for both UniFrac distance matrices (Table 2, Figure S3). These differences in gut region and tank could not be observed graphically in the PCoA since it does not take into account the 100% of the variance (74.77% in weighted UniFrac and 24.01% in unweighted UniFrac) (Figures S2 and S3).

### 3.3. Effect of Allium-Derived PTSO Supplementation on the Body Weight of Gilthead Seabream Juveniles

**Figure 5:**
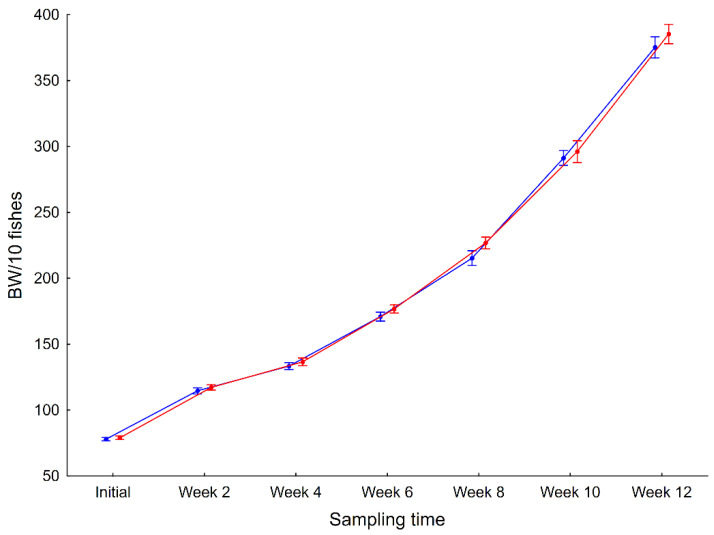
Body weight (g) of control (red) and Allium-derived PTSO (blue) supplemented gilthead bream juveniles during the 12 weeks experiment. Whiskers show ± 95% confidence intervals.

Gilthead seabream juveniles supplemented with PTSO showed similar body weight to that of the control group. Both groups of fish showed a similar trend in body weight throughout the experiment (12 weeks). At the end of the experimental period (12 weeks), no differences in body weight between the control and *Allium*-derived PTSO supplemented group were observed (Table 3, Figure 5).

## 4. Discussion

In this study, the provision of an *Allium*-based product rich in PTSO produced significant changes in the bacterial abundances of some bacterial groups in the foregut and hindgut of gilthead seabream juveniles after 12 weeks of treatment. Differences in PTSO-supplemented fish appeared in both gut regions, with a decrease in potentially pathogenic *Vibrio* and *Pseudomonas* and an increase in beneficial *Lactobacillus* in the foregut and hindgut. These results were accompanied by significant shifts in diversity indices, and no differences in body weight during this experimental period.

Phytogenics modulate gut microbiota, increase productive parameters, appetite stimulation and antipathogenic properties in both terrestrial and aquatic species, resulting in promising functional feed additives [10,52]. Extracts from *Allium* plants, mainly from garlic and onion, have been used as supplement for fish diets in different studies, showing beneficial effects on immune system, growth performance, and health status [53,54]. The effects of these *Allium* extracts are related to secondary metabolites and organosulfur compounds such as allicin, PTS or PTSO [23,24]. Despite the lack of research on the use of PTSO in aquaculture, other organosulfur *Allium*-based compounds have been used as diet supplement in different studies. The addition of allicin on aquafeed showed beneficial effects on the growth performance and survival rate of the large yellow croaker (*Larimichthys crocea*) [55]. Other studies using allicin showed an improvement in biochemical, antioxidant, and immunological parameters of tilapia (*Oreochromis niloticus*) [56], and antibacterial activity in rainbow trout (*Oncorhynchus mykiss*) [57]. However, no studies have yet explored the effects of *Allium*-derived PTSO on the intestinal microbiota of fish species. Our results show an increase in Shannon diversity index and shifts in hindgut microbiota only when minority ASVs were taken into account (unweighted Unifrac). This effect could be related with the in vitro antibacterial, antifungal, and anticoccidial activity of PTSO [25,26,27]. In addition, the influence of PTSO on the intestinal microbiota has been studied in species other than fish. The addition of different doses of PTSO in broiler chickens produced changes in intestinal microbiota, and improved digestibility and productive parameters [32,58]. In laying hens, PTSO supplementation produced an increase in potentially beneficial bacterial genera, in the number of eggs laid, and in egg size [28,30]. In pig production, PTSO also showed beneficial effects in the gut microbiota, and an increase in body weight and productive parameters in both piglets and growing-finishing pigs [31,33].

Our supplementation produced changes in the potential pathogenic genera of the gut microbiota. The relative abundance of *Vibrio* and *Pseudomonas* in the foregut and hindgut significantly decreased in gilthead seabream juveniles supplemented with *Allium*-derived PTSO. *Vibrio* spp. are ubiquitous in marine environments, and some species produce clinical diseases considered to be potentially pathogenic for fish and causing devastating impact [59,60]. Remarkably, other *Allium* extracts have demonstrated antimicrobial activity against this pathogenic species in aquaculture. The supplementation of the diet of Asian seabass (*Lates calcarifer*) with garlic showed an improvement in immunological parameters and survival after a *Vibrio harveyi* challenge [61]. In the same way, the addition of onion to the diet of brown-marbled grouper (*Epinephelus fuscoguttatus*) juveniles reduced susceptibility to *V. harveyi* infection [62]. *Pseudomonas* has also been described as a ubiquitous bacterial genus, although some species such as *Pseudomonas anguilliseptica*, *P. aeruginosa, P. fluorescens,* and *P. putida* are considered emergent opportunistic fish pathogens [63,64,65]. According to our results, several *Allium* extracts also showed antimicrobial activity against *Pseudomonas* species. A study using garlic extract reduced the mortality of tilapia (*O. niloticus*) infected with *P. fluorescens* [66]. Similarly, seabream (*S. aurata*) fed a diet supplemented with a garlic extract presented lower mortality and better therapeutic response to antibiotic treatment when challenged with *P. anguilliseptica* [64].

The supplementation of *Allium*-derived PTSO in the diet of gilthead seabream juveniles also had positive effects, particularly by the increase in potentially beneficial *Lactobacillus* in both foregut and hindgut. This genus is a prevalent constituent of the intestinal microbiota of many fish species and is considered as a beneficial organism associated with a healthy intestinal epithelium and immune system [67,68,69]. Furthermore, some strains can inhibit the adhesion of fish pathogens to the intestinal epithelium [70]. In addition, different studies using *Lactobacillus* as probiotic in aquaculture showed a positive correlation with fish health and productive parameters. The supplementation of probiotic *Lactobacillus* spp. in the live food of gilthead seabream larvae increased digestive enzyme activity and survival [71]. Plant-based diets have been associated with a high abundance of bacteria belonging to the Firmicutes phylum (especially lactic acid bacteria, BAL) in the rainbow trout microbiota [72]. The addition of PTSO also showed an increase in the abundance of different BAL species like *Bifidobacterium, Lactobacillus* and *Lactococcus* in broiler chickens, laying hens and piglets [24]. Similarly, in our study, PTSO supplementation also increased Firmicutes, especially *Lactobacillus*.

Our results show no differences in body weight between control and *Allium*-supplemented fish during the experimental period (12 weeks). Nevertheless, further studies are necessary in order to clarify whether these results can be extrapolated to other growth stages, fish species and under real field conditions. The increase in Shannon diversity index in the foregut and changes in hindgut microbiota in supplemented fish were not correlated with changes in body weight. This result is in accordance with findings in a study with largemouth bronze gudgeon (*Coreius guichenoti*), where differences in bacterial diversity did not translate into differences in body weight [73]. By contrast, some studies demonstrated that changes in alpha diversity could be related with the increase in body weight in birds and with obesity in humans [74,75]. Similarly, Vezza et al. [34] reported that the addition of PTSO in an obesogenic mice model was able to counteract the altered composition and the diversity in the gut microbiota, normalizing the proportion of the major bacteria phyla seen in standard-diet-fed mice, increasing the Shannon diversity index. Furthermore, Büyükdeveci et al. [20] showed an increase in body weight and a decrease in alpha diversity as the garlic concentration increased in the diet of rainbow trout (*O. mykiss*). However, another study on rainbow trout (*O. mykiss*) by Betiku et al. [76] suggested that increased body weight is related to higher bacterial diversity. Although in our study, the general structure of bacterial community did not differ between the control and *Allium*-derived PTSO treatment, we found significant differences in the hindgut when the most abundant ASVs were taken into account. These differences in microbiota could be related with differences in gut morphology and functionality, food processing and nutrient intake [64]. The foregut is the major site of carbohydrate, protein, and lipid digestion, while the absorption of undigested compounds continues in the hindgut [65]. The foregut is closer to the fish’s mouth, and its microbiota could be affected by environmental factors such as diet, water, and other external factors, while the microbiota in the hindgut is more affected by host factors, such as health and genotype [66].

The differences found between tanks were observed in other studies. In a recent study, Minich et al. [77] found a strong association between tank and Atlantic salmon (*Salmo salar*) microbiota in recirculating aquaculture system. There may be a continual bacterial exchange between fish microbiota and the water in the tank [78]. Furthermore, an excess of organic matter in these recirculating aquacultural system tanks, including fish feed and feces, can build up in the tank and promote changes in some bacterial groups [79]. Previous research showed high similarity in fecal microbiota in individuals from the same tank [80]. Fish in the same tank are continuously exchanging excreta and feces, so there is likely a positive feedback between the bacterial community of the gut and the surrounding water, marking strong differences in the gut microbiota between fish from different tanks. Indeed, our supplement produced changes in the majority genera of the intestinal microbiota. The relative abundance of *Vibrio* and *Pseudomonas* in the foregut and hindgut significantly decreased in gilthead seabream juveniles supplemented with *Allium*-derived PTSO.

## 5. Conclusions

Our experimental supplementation of the diet of gilthead seabream juveniles with *Allium*-derived PTSO produced shifts in abundance of the majority bacterial ASVs in the foregut and hindgut, but did not affect growth parameters such as body weight after 12 weeks of experiment. These results are very promising for the use of this phytogenic compound in aquaculture as a potential feed additive that shows a positive effect on the gut microbiota, by reducing potentially pathogenic bacteria such as *Vibrio* and *Pseudomonas* while increasing *Lactobacillus*. However, further research, both at this stage of growth and at later stages, is necessary to study the relationship between these changes found in the microbiota and other parameters related to the health status of fish, such as feed digestibility, intestinal enzyme activity, and the immune system.

## Figures and Tables

**Table 1:** General linear mixed models exploring the effects of the treatment (control and *Allium*-derived PTSO), and gut region as factors, and tank nested in treatment in the different alpha diversity indices of the bacterial community of gilthead seabream juveniles. D.f. refers to degree of freedom. The first number is the degree of freedom of the independent variable, and the second is for the error term. Significant *p*-values (*p* < 0.05) are shown in bold.

	Explanatory Variables	D.f	F	*p*
Bacterial ASV richness	Treatment	1188	3.35	0.386
	Gut Region	1188	3.20	0.075
	Tank (Treatment)	4188	3.56	**0.008**
	Gut Region*Treatment	1188	0.18	0.668
Shannon diversity index	Treatment	1188	20.23	**0.009**
	Gut Region	1188	6.67	**0.011**
	Tank (Treatment)	4188	0.89	0.469
	Gut Region*Treatment	1188	0.91	0.341

**Table 2:** General linear mixed models exploring the effects of treatment, gut region, tank nested in treatment, and the interaction of treatment and gut region in beta diversity indices of the bacterial community of gilthead seabream juveniles fed with control diet or supplemented with *Allium*-derived PTSO. D.f. refers to degree of freedom. The first number is the degree of freedom of the independent variable, and the second is for the error term. Significant *p*-values (*p* < 0.05) are shown in bold.

	β-Diversity Distance Matrix	Explanatory Variables	D.f.	Pseudo-F	*p*
Both gut regions	Weighted UniFrac	Treatment	1195	8.13	0.092
		Gut region	1195	5.13	**0.005**
		Tank (Treatment)	4195	3.31	**0.002**
		Treatment*Gut region	1195	0.44	0.702
	Unweighted UniFrac	Treatment	1195	2.22	0.088
		Gut region	1195	1.65	**0.031**
		Tank (Treatment)	4195	1.83	**0.001**
		Treatment*Gut region	1195	0.83	0.745
Foregut	Weighted UniFrac	Treatment	1195	5.26	0.082
		Tank (Treatment)	4195	2.03	**0.016**
	Unweighted UniFrac	Treatment	1195	1.55	0.189
		Tank (Treatment)	4195	1.38	**0.011**
Hindgut	Weighted UniFrac	Treatment	1195	7.82	0.074
		Tank (Treatment)	4195	2.27	**0.025**
	Unweighted UniFrac	Treatment	1195	2.15	**0.021**
		Tank (Treatment)	4195	1.28	**0.041**

**Table 3:** General linear mixed models exploring the effects of treatment as a factor, sampling time as a continuous factor, and tank nested in treatment as a random factor in the body weight (BW) of gilthead seabream juveniles fed with control diet or supplemented with *Allium*-derived PTSO (average ± SD). D.f. refers to degree of freedom. Sample unit is the body weight of 10 fish. The first number is the degree of freedom of the independent variable, and the second is for the error term. Significant *p*-values are shown in bold.

Sampling Time	Control	*Allium*-Derived PTSO	Independent Variables	F	D.f.	*p*
Mean BW Whole experiment	158.33 ± 5.65	153.84 ± 5.46	Treatment	1.74	1454	0.188
			Tank (treatment)	1.12	4454	0.346
			Sampling time Treatment*sampling time	5438.96 1.85	1454 1454	**<0.001** 0.174
BW Week 12	385.37 ± 3.49	376.32 ± 3.50	Treatment	3.57	1.30	0.057
			Tank (Treatment)	0.35	4.30	0.772

## Data Availability

Sequences are available from the Sequence Read Archive (SRA) at the Genbank—NCBI webpage (https://www.ncbi.nlm.nih.gov/sra, accessed on 23 July 2021), BioProject: PRJNA749674, accession nos. SAMN20396460 to SAMN20396707.

## Supplementary Materials

The following supporting information can be downloaded at https://www.mdpi.com/article/10.3390/ani12141821/s1, Table S1: list of barcodes assigned to each sample for multiplexing and Illumina sequencing. Figure S1: Bar plot summarizing the relative bacterial abundance at the class level in different gut regions (foregut and hindgut) and treatments. Control refers to gilthead seabream juveniles fed with basal diet while *Allium*-derived PTSO refers to experimental gilthead seabream juveniles fed with basal diet supplemented with *Allium*-derived PTSO. Figure S2: principal coordinate analysis based on (A) weighted and (B) unweighted UniFrac distance matrixes exploring differences in bacterial community between both gut regions (blue: foregut, red: hindgut). Percentages show the proportion of variance explained by each axis. Figure S3: principal coordinate analysis based on weighted and unweighted UniFrac distance matrices exploring differences in bacterial gut community among different tanks of control and *Allium*-derived PTSO (red: control, tank 1, blue: control, tank 2, orange: control, tank 3, green: *Allium*-derived PTSO, tank 1, purple: *Allium*-derived PTSO, tank 2, yellow: *Allium*-derived PTSO, tank 3). Percentages show the proportion of variance explained by each axis.
